# Multilevel Framework for Analysis of Protein Folding Involving Disulfide Bond Formation

**DOI:** 10.1021/acs.jpcb.4c00104

**Published:** 2024-03-21

**Authors:** Patryk A. Wesołowski, David J. Wales, Philipp Pracht

**Affiliations:** Yusuf Hamied Department of Chemistry, University of Cambridge, Lensfield Road, Cambridge CB2 1EW, U.K.

## Abstract

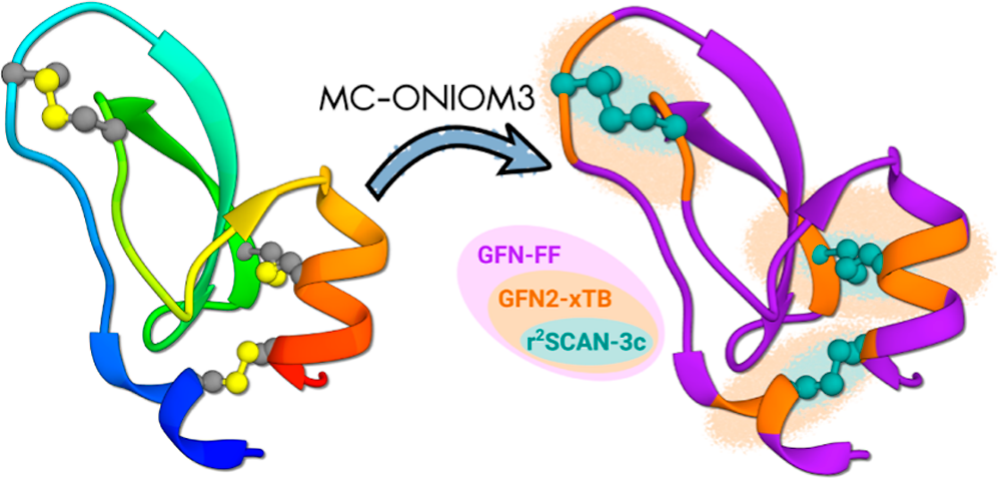

In this study, a three-layered multicenter ONIOM approach is implemented to characterize the naive folding pathway of bovine pancreatic trypsin inhibitor (BPTI). Each layer represents a distinct level of theory, where the initial layer, encompassing the entire protein, is modeled by a general all-atom force-field GFN-FF. An intermediate electronic structure layer consisting of three multicenter fragments is introduced with the state-of-the-art semiempirical tight-binding method GFN2-*x*TB. Higher accuracy, specifically addressing the breaking and formation of the three disulfide bonds, is achieved at the innermost layer using the composite DFT method r^2^SCAN-3c. Our analysis sheds light on the structural stability of BPTI, particularly the significance of interlinking disulfide bonds. The accuracy and efficiency of the multicenter QM/SQM/MM approach are benchmarked using the oxidative formation of cystine. For the folding pathway of BPTI, relative stabilities are investigated through the calculation of free energy contributions for selected intermediates, focusing on the impact of the disulfide bond. Our results highlight the intricate trade-off between accuracy and computational cost, demonstrating that the multicenter ONIOM approach provides a well-balanced and comprehensive solution to describe electronic structure effects in biomolecular systems. We conclude that multiscale energy landscape exploration provides a robust methodology for the study of intriguing biological targets.

## 1 Introduction

Proteins are macromolecular biopolymers essential to the structure and function of carbon-based organisms, governing most biochemical processes within cellular environments. These versatile biomolecules serve as catalysts, facilitate enzymatic reactions, and play important roles in cellular signaling, transport, and structural support. Their optimal functionality depends on a folded native structure. In the past decade, significant scientific advancements, notably through endeavors such as the critical assessment of protein structure prediction (CASP) experiment,^1,2^ showcased remarkable progress in predicting protein conformations. The introduction of machine learning approaches, exemplified by the AlphaFold project,^3,4^ marked a substantial improvement. However, predicting the folding pathway, its associated thermodynamics and kinetics, is different from predicting the folded structure and remains a key ambition in the realms of computational chemistry and biology.^5^ Here, the folding process is a nuanced exploration of conformational space, which may involve thermodynamically stable intermediates and formally leads to the native state on the energy landscape.^6−10^

**Figure 1:**
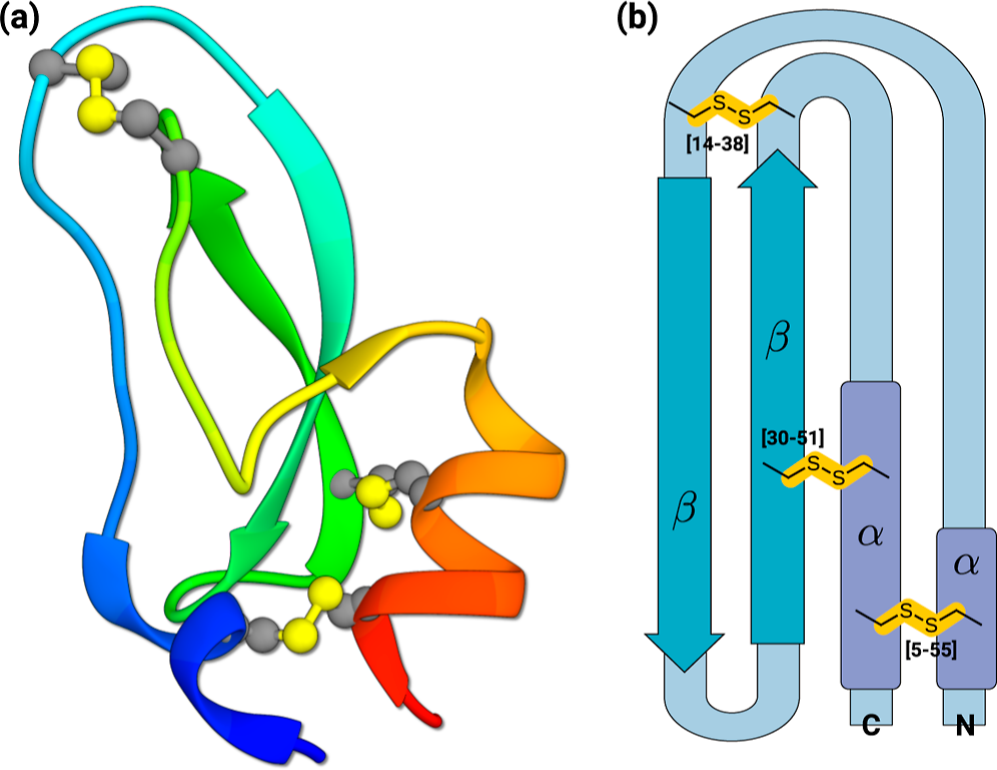
(a) Ribbon model of BPTI from the crystal structure with PDB ID 5PTI.^11^ Cys–Cys sulfide bridges are shown in atomistic representation. (b) Simplified view of the BPTI protein, highlighting key secondary structure motifs and native disulfide bonds.

In this context, one of the most well-studied examples is the analysis of the folding pathway of the bovine pancreatic trypsin inhibitor (BPTI) (Figure 1). BPTI, a monomeric globular polypeptide, includes 16 distinct amino acids with 58 residues folded into a stable and compact tertiary structure. The crystal structure reveals a twisted β-hairpin, C-terminal and N-terminal α-helices, and three disulfide bonds (Cys_30_–Cys_51_, Cys_5_–Cys_55_, Cys_14_–Cys_38_).^11^

The stabilization induced by disulfide bonds in the native state has been thoroughly explored in the literature.^12−15^ The folding pathway energetically favors a structure with a well-defined tertiary arrangement. Intriguingly, the folding pathways encounter intermediate disulfide bonds (Cys_5_–Cys_30_, Cys_5_–Cys_14_, Cys_5_–Cys_38_, and Cys_5_–Cys_51_), absent in the final native state.^14^ BPTI’s modest size and its well-established structural behavior during folding render it an ideal target for scrutinizing the thermodynamical stability of disulfide bonds and their impact on structural stability.

However, achieving high precision in calculations demands ingenuity, posing a significant challenge in the modern computational modeling of proteins.^2,16−18^ Unfortunately, there is a necessary trade-off between computational accuracy and efficiency. The scalability hurdle, where an increase in degrees of freedom leads to a disproportionate rise in computation time, renders quantum mechanical (QM) methods, such as density functional theory (DFT), mostly impractical for large biomolecular systems.^19,20^ Much less costly classical force-field or coarse-grained methods^21^ allow exploration of substantially larger systems but often suffer in terms of accuracy and parameter availability. Balanced computational performance is essential, which in recent years has been addressed by machine learning (ML) methods in materials science^22,23^ and the revival of semiempirical quantum mechanics (SQM).^24,25^

An alternative approach is the construction of multiscale models employing different levels of theory for different parts of the system, and, in particular, QM/MM schemes have emerged as viable for biomolecular systems.^26−29^ Here, chemically important regions of a system, for example, those exerting the greatest influence on structural stability or playing a pivotal role in a reaction, are selected as a “high layer” and assessed with a more accurate method (i.e., QM), while the remaining structure constitutes the “lower layer”, calculated using molecular mechanics (MM) methods.

In this study, we implement a subtractive QM/MM scheme of the popular ONIOM (our own N-layered integrated molecular orbital and molecular mechanics) type^30−34^ to investigate relative structural stability in the BPTI folding pathway. Several studies on BPTI employing various QM/MM models can be found in the literature.^35−37^ Focusing on the formation of the three disulfide bonds, we calculate the initial pathway to the native state with the general all-atom force-field GFN-FF.^38^ A multiscale approach is then used to obtain free energies for selected points of interest along the folding path, employing DFT calculations at the r^2^SCAN-3c level^39,40^ to accurately describe the disulfide bond stability. As a central novelty, we introduce an intermediate SQM level between the DFT and GFN-FF layers to more accurately incorporate electronic structure effects in the vicinity of the cysteine residues. SQM methods of the tight-binding type^24^ have successfully been employed in QM/MM simulations.^41^ Tight-binding models of the GFN*n*-xTB family^25^ have proved to be particularly versatile for exploration of energy landscapes^42−44^ and efficient calculations of thermodynamic properties,^20,45,46^ and an ONIOM methodology has recently been adapted.^25,47^ Our implementation of this multicenter ONIOM (MC-ONIOM) variant extends the approach, promising even greater computational time savings for large systems.^34^

We first present the multicenter and multilayer ONIOM approach, followed by a discussion on the simplest dipeptide containing a disulfide bond, cystine, to validate the theory behind MC-ONIOM for BPTI, followed by a discussion of the folding pathway. All the benchmarking calculations were performed in vacuum. This approach avoids additional complications associated with the choice of a solvent model (explicit, implicit, or a hybrid of both) and associated sampling issues. Further, the choice of suitable implicit solvation models within the ONIOM context can be especially challenging and sometimes requires dedicated implementations.^32^ We plan to extend our tests to include solvation in future work. The constraints associated with the disulfide bond network enable us to focus on well-defined events on the folding pathway, which is a particular advantage for a benchmarking effort. We aim to gain insights on the role of disulfide bond stabilities in the BPTI folding pathways and highlight the potential shortcomings of modeling these molecular rearrangements with classical force-field methods.

## 2 Theory and Technical Details

### 2.1 Multicenter *n*-Layer ONIOM

**Figure 2:**
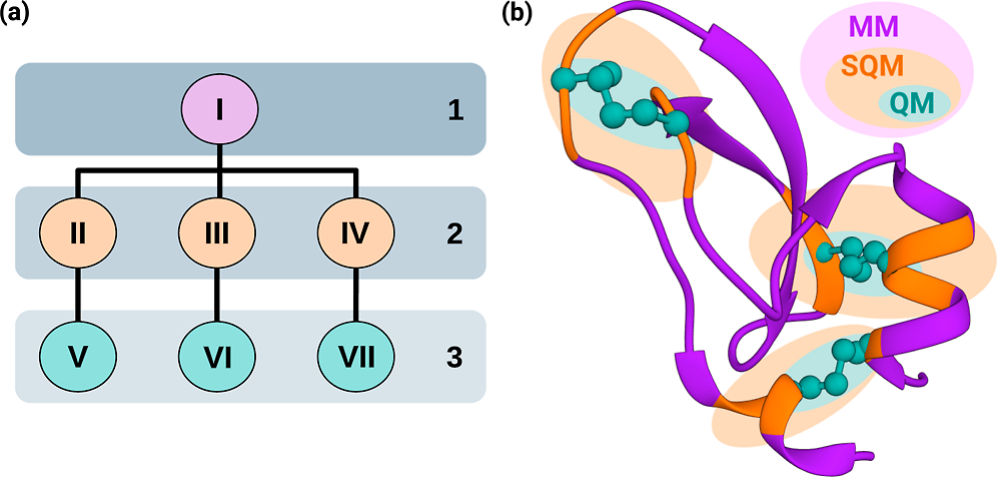
(a) Scheme detailing the construction of a three-layer MC-ONIOM dependency tree. Nodes in the diagram correspond to fragments, with the primary layer (layer 1) requiring a solitary node encompassing all system atoms. Subsequent layers (layers 2 and 3) have the flexibility to encompass various nonoverlapping subsystems, functioning as child nodes of the initial node or nodes in subsequently higher layers. (b) Three-layer three-center ONIOM(r^2^SCAN-3c:GFN2-xTB:GFN-FF) partitioning of the BPTI protein. Each ONIOM center is built around one Cys–Cys sulfide bridge.

We have implemented a multicenter *n*-layer ONIOM mechanical embedding method, closely following the approach presented by Seeber et al.^34^ In this context, the ONIOM layer dependencies can be most effectively illustrated as a tree graph, as exemplified in Figure 2a.

Each node corresponds to a substructure of the original system and, with the exclusion of the initial structure, is explicitly linked to a parent node contingent upon its layer. The user is required to allocate atoms from the initial system to different nodes, with truncated bonds being automatically saturated through the linking atoms.

By statically placing the saturating link atom at **r**_*l*_′ along the vector of the cut bond **r**_*a*_ – **r**_*b*_, we avoid introducing any additional degrees of freedom
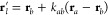
1The appropriate value for the factor *k*_*ab*_ is determined by evaluating the ratio of the covalent radii^48^ (*R*^cov^) of the relevant atoms using
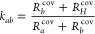
2where the linking atoms are specified as hydrogen, and thus, *R*_*H*_^cov^ represents the covalent radius of hydrogen.

The final dependency tree facilitates the recursive assembly of the overall ONIOM properties. The general expression for building the energy, gradient, or Hessian, of a specific node, represented as , is given by
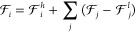
3Here,  denotes the construction of the high-level energy/gradient for the parent node, *F*_*j*_ represents the recursively formed property of the child nodes, and  signifies their contribution to the low-level energy/gradient/Hessian. It is essential to note that the high-level calculations for each parent node align with the same level of theory as the low-level calculations for its respective child nodes. The recursion concludes when a node no longer has additional child nodes, at which point . The gradient of the *i*-th model system **g**_*i*_′ can be projected into the basis of the real system **g**_*i*_ via
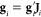
4

In a similar fashion, the Hessian matrix of the individual fragments **H**_*i*_′ can be projected into the basis of the real system via
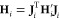
5

The Jacobian employed in both eqs 4 and 5 is given by
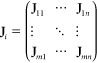
6where *m* is the dimension of the *i*-th subsystem or fragment, and *n* is the dimension of the real system. The corresponding matrix elements **J**_*nm*_ are given by
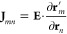
7where **E** is a 3 × 3 identity matrix. The derivatives  are either 1, 0, or a value depending on *k*_*ab*_ due to eq 1. Once all gradients (or Hessians) of the subsystems have been projected into the basis of the real system, constructing the full MC-ONIOM*n* gradient (or Hessian) is possible via the recursive algorithm given by eq 3.

In this project, we employ a three-layer MC-ONIOM to investigate structures sampled from the BPTI folding pathway. The corresponding multiple centers are illustrated in Figure 2b, where each layer corresponds to a distinct level of accuracy. The initial layer provides the assessment of the entire structure at a classical force-field level, a task accomplished using GFN-FF.^38^ The second, intermediate layer comprises three multicenter fragments, each addressed with GFN2-xTB.^49^ The third and final layer includes only the three Cys–Cys sulfide bridges and is computed using r^2^SCAN-3c.^39,40^

### 2.2 Technical Details

The MC-ONIOM approach was implemented in a standalone Fortran library called lwONIOM, which is freely available under the LGPL-3.0 license from GitHub.^50,51^ The library provides bookkeeping and ONIOM partitioning functionalities (cf. Section 2.1) for mechanical embedding, but adhering to the eponymous “light-weight” in the coding of lwONIOM, no potential calculators (energies and gradients) are implemented at the time of writing. To provide this capability, lwONIOM was interfaced in the recently developed CREST program.^42,51,52^ r^2^SCAN-3c DFT calculations were performed with the ORCA program package.^53,54^ SQM and MM calculations were conducted with dedicated libraries implemented in the CREST program, employing GFN2-xTB^25,49^ and GFN-FF^25,38^ methodologies, respectively. If not stated otherwise, we will refer to r^2^SCAN-3c^39,40^ as “DFT”, to GFN2-xTB^49^ as “SQM”, and to GFN-FF^38^ as “FF”. In particular, two-layer ONIOM(DFT:SQM) and three-layer ONIOM(DFT:SQM:FF) are assessed with regard to their performance and computation times. Following standard conventions,^32,34^ these approaches are denoted as (MC−)ONIOM*n*, with *n* referring to the number of layers. These levels of theory were first evaluated for the formation of cystine, where we compared the reaction energies (Δ*E*) and free energies at 298.15 K (Δ*G*^298.15^). For reference, high-level DFT calculations were conducted with the range-separated ωB97X-V functional^55^ employing a polarized quadruple-ζ basis set def2-QZVPP.^56^ All DFT calculations in this study were conducted using the *TightSCF* and *DefGrid3* settings in ORCA. Calculation of the free energy employed the modified rigid-rotor-harmonic-oscillator (mRRHO) approximation for δ*G*_mRRHO_ contributions, using Grimme’s rovibrational entropy interpolation for frequencies less than 25 cm^–1^.^57,58^ The corresponding calculations were performed with the CREST code.

For BPTI, CREST was first employed to prepare the unfolded linear state and optimize both the unfolded and native structures. The OPTIM program,^59^ interfaced with GFN-FF, was used to facilitate the calculation of the pathway between the unfolded and native states. Importantly, all pathway calculations for BPTI associated with the GFN-FF level of theory employed the same reference topology of the native state to enable the correct cleavage and (re)formation of disulfide bonds since the a priori creation of new bonds is not possible in the current formulation. The geometry optimization techniques employed have been reviewed before.^6,7,60^ Very briefly, transition state (TS) candidates are obtained using the doubly-nudged^61,62^ elastic band^63−66^ (DNEB) method. We first obtained an initial database of stationary points by optimizing all structures from an approximate pathway obtained via quasi-continuous interpolation (QCI).^67,68^ The collected minima were then connected pairwise via DNEB, employing up to 150 images each, and TS candidates and new minima were added back to the database, from which a complete pathway was identified. From this pathway, we selected the TS and associated minima describing the three disulfide bond cleavages. These minima were subjected to a detailed analysis of their relative stabilities using the MC-ONIOM approach to calculate free energy corrections from the reconstructed (eqs 3 and 5) Hessian within the mRRHO approximation, as described above. For simplicity, all calculations were originally performed in the gas-phase, as explained in the Introduction. Again, since one goal is to highlight the computational methodology, referring to vacuum calculations removes one layer of complexity (choice of solvent model, parametrization, etc.) and allows us to focus on the calculation strategy. However, where feasible, the gas-phase results are supplemented by calculations performed with GFN-FF and the ALPB implicit solvation model^69^ for water.

## 3 Results and Discussion

### 3.1 Cystine

**Figure 3:**
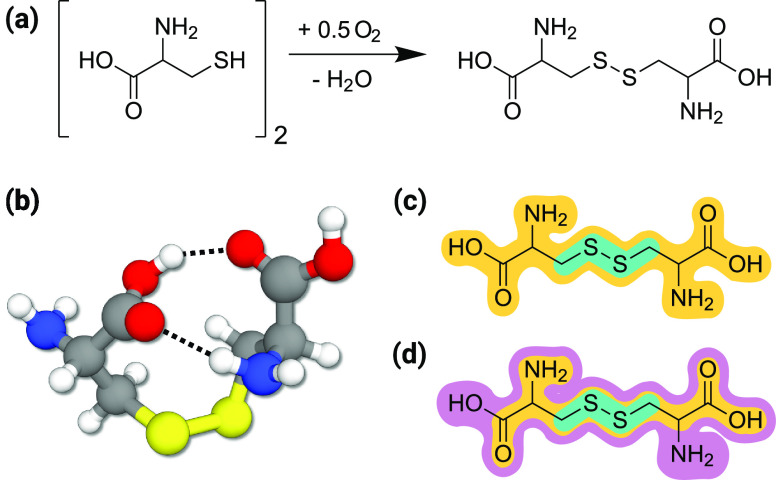
(a) Formation reaction of cystine from two cysteine monomers. (b) Most favorable gas-phase conformer of cystine, calculated at the GFN-FF level. (c) Schematic two-layer ONIOM(r^2^SCAN-3c:GFN2-xTB) setup. (d) Schematic three-layer ONIOM(r^2^SCAN-3c/GFN2-xTB:GFN-FF) setup.

Initially, we benchmarked our methodology on a simple biomolecular system containing a disulfide bond, specifically the cystine dipeptide. The dimeric structure is formally created by the oxidative reaction of two cysteine monomers.^70^ Calculations of Δ*E* and Δ*G*^298.15^ were conducted by following the reaction outlined in Figure 3a, employing various levels of accuracy: GFN2-xTB, GFN-FF, ONIOM(DFT:SQM), and ONIOM(DFT:SQM:FF), as well as r^2^SCAN-3c itself, and a high-accuracy ωB97X-V/def2-QZVPP//r^2^SCAN-3c level.

Table 1 presents a thorough examination of gas-phase reaction energies (Δ*E*), free energies at 298.15 K (Δ*G*^298.15^), and computational efficiency across diverse methods applied to assess the oxidation of two cysteine monomers to cystine (cf. Figure 3a). The reference method, ωB97X-V/def2-QZVPP//r^2^SCAN-3c, establishes a baseline with a Δ*E* of −75.00 kcal mol^–1^ and Δ*G*^298.15^ of −62.87 kcal mol^–1^. The range-separated ωB97X-V functional,^55^ known for its performance in established benchmark databases like GMTKN55,^71,72^ serves as an excellent reference and is crucial for evaluating alternative methods. Unfortunately, its high computational cost renders it unsuitable for use in large-scale ONIOM setups requiring hundreds of energy and gradient evaluations. r^2^SCAN-3c, when compared to the ωB97X-V reference, exhibits a closely aligned Δ*E* of −78.31 kcal mol^–1^, a Δ*G*^298.15^ of −65.97 kcal mol^–1^, and a balanced computation time, making it a viable choice within the ONIOM scheme. Furthermore, r^2^SCAN-3c serves as the level for the geometry optimization of the reference method. Frequencies calculated with ωB97X-V/def2-QZVPP for these structures were checked for the absence of imaginary modes, confirming that this was an adequate choice.

**Table 1:** Gas-Phase Reaction Energies (Δ*E*), Free Energies at 298.15 K (Δ*G*^298.15^), and Cumulative CPU Times per Singlepoint Calculation for the Oxidation of Two Cysteine Monomers to Cystine (cf. Figure 3A), Calculated at Several Levels of Theory^a^

method	CPU time [s]	Δ*E* [kcal mol^–1^]	Δ*G*^298.15^ [kcal mol^–1^]
GFN-FF	0.03	–17.51	–2.21
GFN2-xTB	0.09	–95.28	–81.98
ONIOM3(DFT:SQM:FF)	19.83	–91.81	–78.03
ONIOM2(DFT:FF)	19.92	–101.23	–86.60
ONIOM2(DFT:SQM)	20.10	–85.69	–72.49
r^2^SCAN-3c	49.08	–78.31	–65.97
ωB97X-V/def2-QZVPP//DFT	731.88	–75.00	–62.87

a DFT refers to r^2^SCAN-3c, SQM refers to GFN2-xTB, and FF refers to GFN-FF.

A noteworthy observation is the computational efficiency of the GFN-FF method, with a CPU time of 0.03 s. However, this efficiency comes with a trade-off, with differences in Δ*E* (−17.51 kcal mol^–1^) and Δ*G*^298.15^ (−2.21 kcal mol^–1^) from the reference. These observations are expected for a classical force-field method. Nonetheless, with a δ*G*_mRRHO_ of approximately 15.30 kcal mol^–1^, GFN-FF demonstrates its suitability^46^ to calculate free energy contributions, which are on the same order of magnitude as for the higher-level reference methods. In contrast, the semiempirical electronic structure method GFN2-xTB achieves a substantially better Δ*E* of −95.28 kcal mol^–1^ and Δ*G*^298.15^ of −81.98 kcal mol^–1^, albeit for a longer CPU time of 0.09 s per singlepoint calculation. The differences in the computational cost of GFN2-xTB and GFN-FF are insignificant for the cystine model system but can become an important factor for larger biomolecules like BPTI.

The ONIOM3(DFT:SQM:FF) and ONIOM2(DFT:SQM) methodologies exhibit intermediate results for Δ*E* and Δ*G*^298.15^ when compared to GFN2-xTB and the DFT references. Specifically, ONIOM3 yields values of −91.81 and −78.03 kcal mol^–1^ for Δ*E* and Δ*G*^298.15^, while for ONIOM2, these values were −85.69 and −72.49 kcal mol^–1^, respectively. Omission of the SQM “middle” layer, i.e., using an ONIOM2(DFT:FF) approach, does not improve on ONIOM2(DFT:SQM) nor ONIOM3, and strongly overestimates the cystine stability, with an Δ*E* of −101.23 kcal mol^–1^, and Δ*G*^298.15^ of −86.60 kcal mol^–1^. Additionally, all three calculations have a similar computational cost of around 20 s of CPU time per singlepoint calculation. Hence, these methods strike a balance between accuracy and computational cost but ultimately correlate with the level of theory. The seemingly superior accuracy of ONIOM2 compared with ONIOM3 can be attributed to two factors: ONIOM3 incorporates less accurate GFN-FF results, impacting overall precision, and the choice of ONIOM subsystems proves suboptimal for ONIOM3, especially regarding different setups for the two cysteine units. Essentially, the ONIOM3 setup omits important regions of noncovalent interactions and thus introduces additional errors in the cystine test case. This is a well-known challenge for QM/MM schemes, particularly for calculations within the supermolecular approach.^32,33^ In the context of BPTI, this result demonstrates that regions close to the disulfide bonds must be described at the same level to avoid truncating noncovalent interactions. Therefore, in the following section, the cysteine units will be described at the DFT level, while all adjacent residues are modeled with GFN2-xTB. The remaining residues are modeled by the low-level force-field.

In summary, while GFN-FF excels in computational efficiency, ONIOM schemes strike a useful balance between accuracy and efficiency. The discrepancy between ONIOM2 and ONIOM3 underscores the significance of methodological considerations, including the choice of subsystems, in achieving accurate results. Compared with the reference method ωB97X-V/def2-QZVPP, r^2^SCAN-3c proves to be a suitable choice for the ONIOM high-level calculations, providing reliable and cost-efficient Δ*E* and Δ*G*^298.15^ values.

### 3.2 BPTI

**Figure 4:**
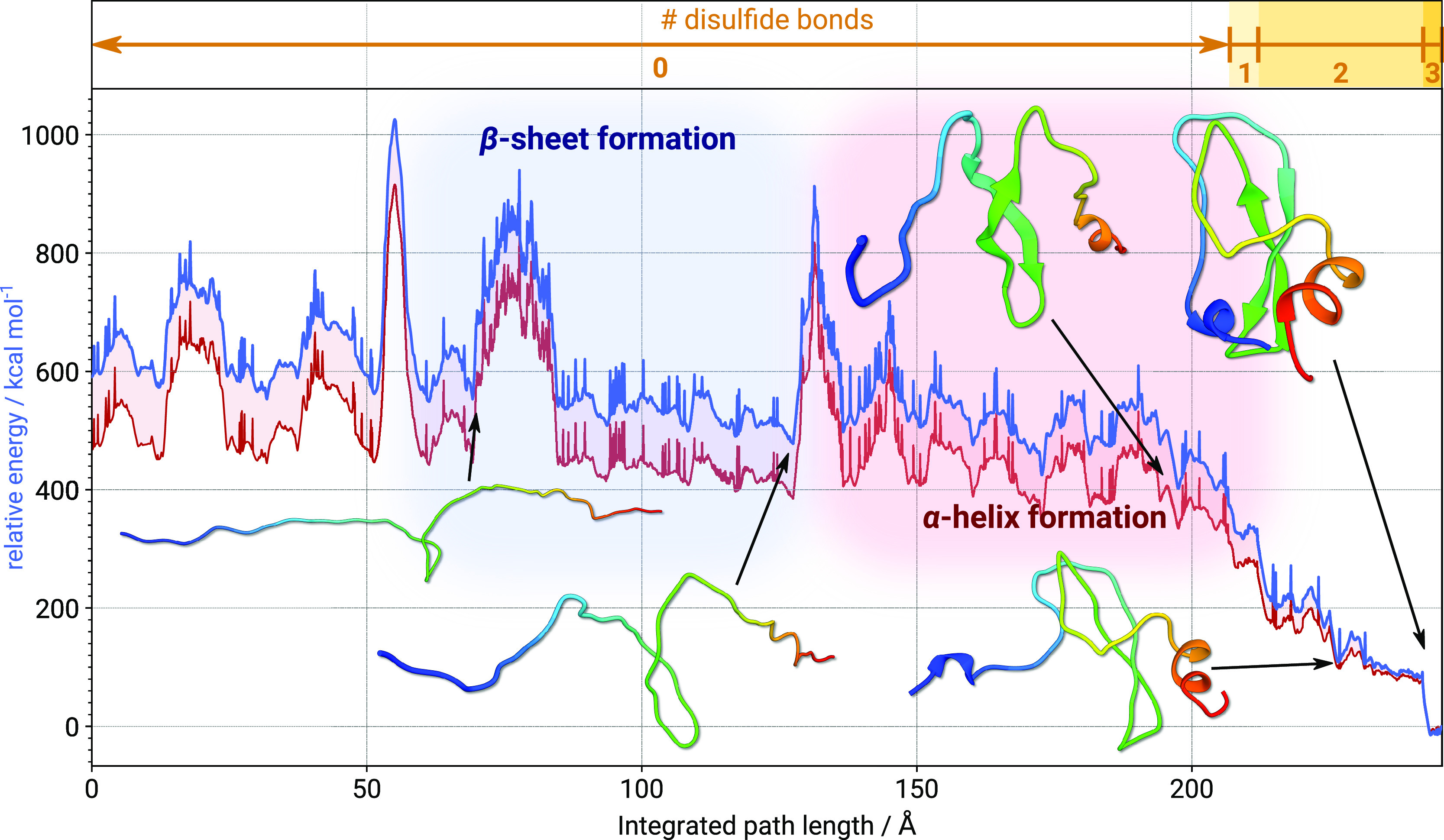
Folding pathway (in blue) for the BPTI protein, showing the energy scale on the left and the number of disulfide bonds along the path length (in yellow) on top. The second energy curve plotted in red corresponds to singlepoint energy evaluations of the gas-phase folding pathway re-evaluated with ALPB(H_2_O) implicit solvation. Sections of the pathway where the formation of the β-sheet and α-helix motif occurs are shaded in blue and red, respectively.

Before studying the disulfide bond stability via an ONIOM approach, we employed GFN-FF in conjunction with the OPTIM program to elucidate the folding pathway of BPTI. GFN-FF showcases robustness over a broad variety of chemical systems,^38^ making it well-suited for applications involving elements up to radon, *Z* ≤ 86. A limitation arises in the inability of GFN-FF to represent the formation of new bonds. Nevertheless, GFN-FF, functioning as a dissociative force-field, can model the homolytic rupture and reformation of disulfide bonds, which was exploited for the pathway construction using the DNEB approach (§2.2). This approach necessitates a formal force-field setup from the unfolding of the native state employing the native topology, i.e., containing the disulfide bonds, in all the calculations. The corresponding pathway is shown in Figure 4.

**Figure 5:**
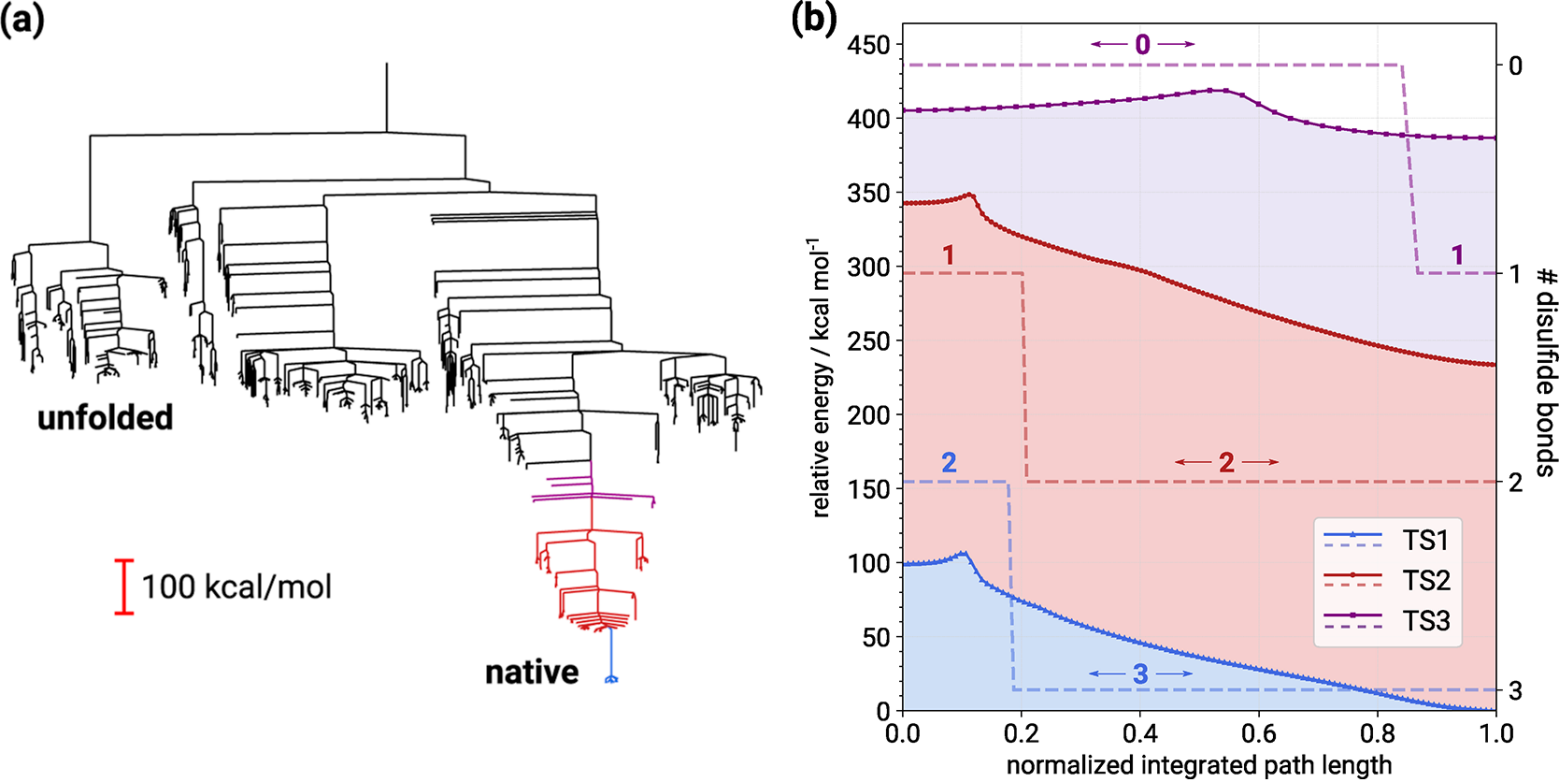
(a) Disconnectivity graph at the GFN-FF level, corresponding to the path in Figure 4. (b) Pathways for the homolytic cleavage of the Cys_5_–Cys_55_ (TS1), Cys_30_–Cys_51_ (TS2), and Cys_14_–Cys_38_ (TS3) disulfide bonds, calculated at the GFN-FF level. Energies are shown relative to the starting point minimum of the TS1 pathway.

The integrated path length is approximately 250 Å, with the most pronounced energetic stabilization occurring in the final third of the pathway, coinciding with the formation of the disulfide bonds. We note at this point that local minima along the pathway may differ with and without consideration of solvation effects. However, since the DNEB approach starts from two defined endpoints (i.e., the unfolded and native state of BPTI) and the initial interpolation does not know about the “true” potential, we can reasonably expect that the overall folding trajectory will look similar. Nonetheless, large effects on the energetics must be expected upon the inclusion of a solvent. Evaluation of the same pathway at the GFN-FF/ALPB(H_2_O) level of theory, therefore, reveals typical effects of implicit salvation: Generally, implicit solvation attenuates noncovalent interactions,^45^ which in this case manifests in an overall lower Δ*E* between folded and unfolded conformations, i.e., the relative energies in implicit solvation are lower compared to the gas-phase energies in Figure 4. Furthermore, unfolded conformations are more strongly affected by implicit solvation due to their larger surface area (to which part of the implicit solvation energy is proportional^69^). Secondary structure folding events commence with the formation of the β-sheet, succeeded by the development of the C-terminal and N-terminal helixes, respectively. This hierarchy is reflected by the disconnectivity graph^73,74^ for the stationary point database corresponding to the pathway, which is shown in Figure 5a. Here, distinct funnels for the secondary motifs and the main funnel containing the disulfide bond formation are clear. The disconnectivity graph in Figure 5a is colored to highlight the pathways leading to the creation of subsequent disulfide bonds. These sections of the pathway are associated with the three transition states TS1, TS2, and TS3, in order of unfolding, where the Cys_5_–Cys_55_ disulfide bond makes or breaks in TS1, Cys_30_–Cys_51_ in TS2, and Cys_14_–Cys_38_ in TS3. Magnified sections of the corresponding pathways of TS1, TS2, and TS3 are shown in Figure 5b, with the energy origin given relative to the starting minimum of the TS1 pathway.

Due to the technical setup of the DNEB method, our pathway deviates from the sequence of disulfide cleavage events outlined in the literature.^14,75^ Darby et al. proposed a pathway where the Cys_30_–Cys_51_ bond forms first, followed by some alternative Cys–Cys combinations, Cys_5_–Cys_55_, and finally Cys_14_–Cys_38_. On the other hand, while our calculations agree that Cys_30_–Cys_51_ (TS2) forms before Cys_5_–Cys_55_ (TS1), Cys_14_–Cys_38_ (TS3) forms first in our calculation, while it forms last according to the (most probable) experimentally observed pathway. The latter effect is a product of using only a single DNEB interpolation, which first “curls” the backchain of the protein before bringing the N and C termini closer together (cf. Figure 4). These results demonstrate the technical capabilities of the DNEB approach in combination with GFN-FF. For the purpose of computational efficiency, we focus on this single pathway to study the relative disulfide bond stability in terms of benchmarking. If the pathway is broken into smaller sections and alternative endpoints are explored, this approach can be adapted for the reproduction of competing disulfide bond formation from the experiment. Unfortunately, the force-field setup prevents us from exploring the alternative pathways that lead to the formation of non-native disulfide bond intermediates, such as Cys_5_–Cys_14_ and Cys_5_–Cys_38_. Since the corresponding disulfide bonds are not present in the topology, any force between these sulfur atoms at a close distance would be strongly repulsive. It is, of course, possible to model the individual pathways via standalone DNEB calculations, but connecting them on a single energy surface will require some code modifications to the force-field to allow manual definition of possible bonds. Nonetheless, previous studies indicate that the rearrangement process is unaffected by non-native disulfide bonds, as they tend to occur in relatively unfolded regions of the molecule.^75^ The corresponding bonds are not deemed “committed” to the rearrangement process, suggesting that they form after the rate-determining step.^76^ Hence, we believe that our example folding pathway is still an adequate representation to showcase the DNEB and MC-ONIOM*n* methodologies and for judging the relative disulfide bond stability via a supramolecular approach.

Concerning the overall disulfide bond stability, the primary question is the physical nature of the GFN-FF pathway. In fact, some problems may result due to the construction of the GFN-FF potential: The actual cleavage of bonds has no barrier in GFN-FF since the binding potential is modeled with a Gaussian function.^38^ The transition states shown in Figure 5b therefore correspond to the first conformational rearrangement after the disulfide bond cleavage, rather than the bond breaking itself. In essence, the pathway lacks physical validity due to the aforementioned homolytically broken disulfide bonds. Formally, the cleaved cysteine residues are treated as radicals, constituting a chemical misrepresentation. In an aqueous environment, cysteine residues, contrary to this assumed behavior, exhibit disulfide formation more closely aligned with the oxidative reaction associated with the cystine formation, as modeled in Section 3.1. In a biological context, the corresponding reaction is driven by disulfide isomerase.^77^ Consequently, the cleaved disulfide bonds necessitate the saturation of sulfur atoms with hydrogen to form thiols. To faithfully represent this scenario, reaction-free energies Δ*G*, as employed previously for cystine, are utilized and further elucidated in a supramolecular approach. The supramolecular approach enables us to focus on differences in relative stability rather than pathways that might anyway lack physical validity.

Within the broader context of our study, we focus on the significance of the disulfide bond formation through modeling homolytic cleavage at the GFN-FF level. Our findings suggest a useful model featuring three distinct transition states associated with significant barriers along the overall pathway. Qualitatively, the disconnectivity graph of Figure 5a suggests an energy difference between the native (NS) and homolytically unfolded (UF) states of around 600 kcal mol^–1^. The disulfide bond funnel alone contributes approximately 400 kcal mol^–1^, constituting roughly two-thirds of this energy difference. Clearly, the formation of these bonds is expected to contribute significantly to the stability of folded BPTI. Table 2 presents the reaction energies (Δ*E*) and free energies at 298.15 K (Δ*G*^298.15^) for three disulfide bond cleavages, calculated using MC-ONIOM3(DFT:SQM:FF) and GFN-FF.

**Table 2:** Reaction Energies (Δ*E*) and Free Energies at 298.15 K (Δ*G*^298.15^) for the Three Disulfide Bond Cleavages Calculated with MC-ONIOM3(DFT:SQM:FF) and GFN-FF^a^

	MC-ONIOM3		GFN-FF (sat.)		GFN-FF (unsat.)	
states	Δ*E* [kcal mol^–1^]	Δ*G*^298.15^ [kcal mol^–1^]	Δ*E* [kcal mol^–1^]	Δ*G*^298.15^ [kcal mol^–1^]	Δ*E* [kcal mol^–1^]	Δ*G*^298.15^ [kcal mol^–1^]
TS1	55.64	63.57	–10.88	–5.79	91.64	90.26
TS2	34.30	30.93	–28.99	–25.95	99.90	80.64
TS3	36.45	47.85	–56.52	–41.46	17.76	17.79
∑	126.38	142.35	–96.40	–73.20	209.30	188.69
NS/UF	628.92	410.46	356.19	314.56	565.98	492.88

a The ONIOM calculation and the GFN-FF(sat.) reaction refer to formal oxidation, as for cystine in Figure 3a, GFN-FF(unsat.) refers to the pathways from Figure 5B with homolytic disulfide bond cleavage.

In evaluating the reactions within the MC-ONIOM3 framework, both the changes in energy (Δ*E*) and the corresponding Δ*G*^298.15^ align with expected values for disulfide bond cleavages. As evidenced by comparisons with cystine (cf. Section 3.1), both reaction energies and free energy contributions are of the correct order of magnitude, attributing to their physical plausibility. The three transition states corresponding to disulfide bond formation/cleavage, TS1, TS2, and TS3, exhibit Δ*E* values of 55.64, 34.30, and 36.45 kcal mol^–1^, respectively, while the corresponding Δ*G*^298.15^ values are 63.57, 30.93, and 47.85 kcal mol^–1^. The stability of the first disulfide bond, Cys_5_–Cys_55_, is notably greater than the subsequent values for TS2 and TS3. One rationale for this observation can be attributed to the influence of noncovalent interactions. TS1 is positioned “early” in our simulation of the cleavage pathway, exhibiting a predominantly folded configuration that requires disruption of numerous noncovalent before bond rupture. Further rotational barriers, particularly linked to α and β motifs, exert a stabilizing influence through intramolecular noncovalent interactions. As a result, although the representation of disulfide bonds may account for part of the energy difference, the overall structural stability results from a combination of multiple contributing factors. Obviously, a free energy difference cannot be compared directly to observable rates. However, through experimental measurements, it was confirmed that the quasi-native (Cys_5_–Cys_55_, Cys_14_–Cys_38_) intermediate is very stable, whereas the Cys_30_–Cys_51_ conformation requires recombination and the formation of non-native disulfide bonds first.^75^ We see this observation qualitatively confirmed by the relative stabilities calculated via the supramolecular approach and MC-ONIOM3: TS1 (Cys_5_–Cys_55_) has the largest Δ*G*^298.15^, followed by TS3 (Cys_14_–Cys_38_), while TS2 (Cys_30_–Cys_51_) is the least stable. Furthermore, TS3 was observed to form rapidly,^14,75^ which is consistent with the assignment of the smallest activation barrier among the three cleavages (cf. Figure 5b).

A discrepancy arises for GFN-FF following the same (chemically correct) supramolecular approach, as the sign of the reaction energies is incorrect. This inconsistency is expected and underlines the intrinsic limitations of force-field methods in capturing the intricacies of chemical reactions. However, an interesting observation emerges when disulfide bond cleavage is approached homolytically, surprisingly revealing a correct sign. Here, the stabilities of TS1 and TS2 are overestimated compared to the underestimated TS3, indicating an overstabilization due to noncovalent interactions within the force-field. Nonetheless, energies and free energies are on a similar scale to MC-ONIOM3 and differ only by a factor of two to three. The cumulative values for the three reactions are also provided, emphasizing the substantial differences in energy and free energy between the methodologies and defining the overall importance of the disulfide bonds. For MC-ONIOM3, the cumulative energy contribution contributes to roughly 20.1% of the total NS/UF difference and approximately one-third (33.9%) of the free energy difference. The homolytically cleaving GFN-FF calculations estimate the same contributions as 37.0% and 38.3%, respectively. These results clearly demonstrate the relevance of the disulfide bonds for the BPTI folding process, although GFN-FF, again, qualitatively overestimates the corresponding contribution. Overall, the results presented in Table 2 underscore the importance of the chosen computational approach in determining the correct energetics of disulfide bond cleavages and emphasize the shortcomings of classical force-fields. Deliberately modeling a chemically implausible pathway, i.e., with homolytic disulfide bond ruptures, may provide a useful computational strategy to model the folding process. However, we strongly recommend investigating this possibility on a case-by-case basis. The ONIOM framework provides a useful strategy to obtain accurate energetics at a lower-than-DFT cost. In particular, the Hessian recombination according to eq 5 provides great computation time savings for Δ*G* calculations. Here, the seminumerical calculation at the GFN-FF level took 10 min 11 s, while the corresponding MC-ONIOM3 calculation took 146 min 32 s (both on 10 CPUs of the same type). The latter value is identical to the accumulation of all ONIOM subsystem Hessian calculations and, therefore, is much cheaper than even a singlepoint energy of the entire system at the DFT level, which we were unable to obtain within a 24 h job time limit.

As noted above, the inclusion of solvent effects can strongly influence the calculated energetics. Therefore, reaction (free) energies were also calculated by employing GFN-FF with the ALPB(H_2_O) implicit solvation model and are given in Table 3. For the respective MC-ONIOM3 calculations, only the GFN-FF layer experiences the implicit solvation potential. The efficacy of this approach will be investigated in future studies; however, as an approximate treatment, it is sufficient to at least qualitatively capture some of the solvation effects.

**Table 3:** Reaction Energies (Δ*E*) and Free Energies at 298.15 K (Δ*G*^298.15^) for the Three Disulfide Bond Cleavages Calculated with MC-ONIOM3(DFT:SQM:FF/ALPB(H_2_O)) and GFN-FF/ALPB(H_2_O)^a^

	MC-ONIOM3		GFN-FF/ALPB (sat.)		GFN-FF/ALPB (unsat.)	
states	Δ*E* [kcal mol^–1^]	Δ*G*^298.15^ [kcal mol^–1^]	Δ*E* [kcal mol^–1^]	Δ*G*^298.15^ [kcal mol^–1^]	Δ*E* [kcal mol^–1^]	Δ*G*^298.15^ [kcal mol^–1^]
TS1	75.97	79.95	1.92	5.52	39.09	40.18
TS2	59.20	33.03	–15.07	–0.54	43.37	37.26
TS3	34.81	57.00	–37.50	–20.89	25.29	33.93
∑	169.97	169.98	–50.65	–15.91	107.75	111.36
NS/UF	429.42	371.61	281.58	235.96	413.22	350.56

a The ONIOM calculation and the GFN-FF(sat.) reaction refer to formal oxidation, as for cystine in Figure 3a, GFN-FF(unsat.) refers to the pathways from Figure 5b with homolytic disulfide bond cleavage. Within the ONIOM calculation, ALPB implicit solvation was employed only for the outer force-field layer.

Generally, the energy difference between the folded and unfolded states of BPTI is reduced due to implicit solvation. As noted earlier, this effect is due to an attenuation of noncovalent interactions and a lowering of the unfolded state energies relative to the native state, presumably due to the large solvent-accessible surface area. For MC-ONIOM3, energy differences for TS1 and TS2 are slightly greater and for TS3 slightly lower than the corresponding gas-phase calculations. Free energy contributions for TS1 and TS3 further stabilize the corresponding disulfide bonds (i.e., Δ*G*^298.15^ is greater than the respective Δ*E*) but destabilize TS2. These results are again qualitatively in line with experimental^75^ observations: The large Δ*G*^298.15^ for TS1 and TS3 can explain the formation of a very stable quasi-native (Cys_5_–Cys_55_, Cys_14_–Cys_38_) structure, while the Cys_30_–Cys_51_ is a partly folded intermediate and thus should exhibit a smaller free energy difference for the disulfide bond formation. On the other hand, calculations performed at the GFN-FF/ALPB(H_2_O) (sat.) level once more exhibit incorrect signs for TS2 and TS3, further highlighting the limitations of the force-field method when applied in supramolecular approaches. Similarly, the artificial homolytic cleavage structures calculated at the same level of theory again provide estimates surprisingly close to those of the reference MC-ONIOM3 approach. The contribution of disulfide bond stability to the NS/UF difference is significantly larger (≈45% for Δ*G*^298.15^ at the MC-ONIOM3 level) than in the gas-phase. This result underscores the critical role of covalent disulfide bonds relative to the dampened noncovalent interactions of the protein in solution. Solvent effects play a crucial role in assessing this stability. However, considering that these effects are most pronounced in the unfolded regions of the pathway (cf. Figure 4) rather than in the regions where disulfide bonds form, we will revert to vacuum calculations in subsequent discussions to bypass further complexities.

**Figure 6:**
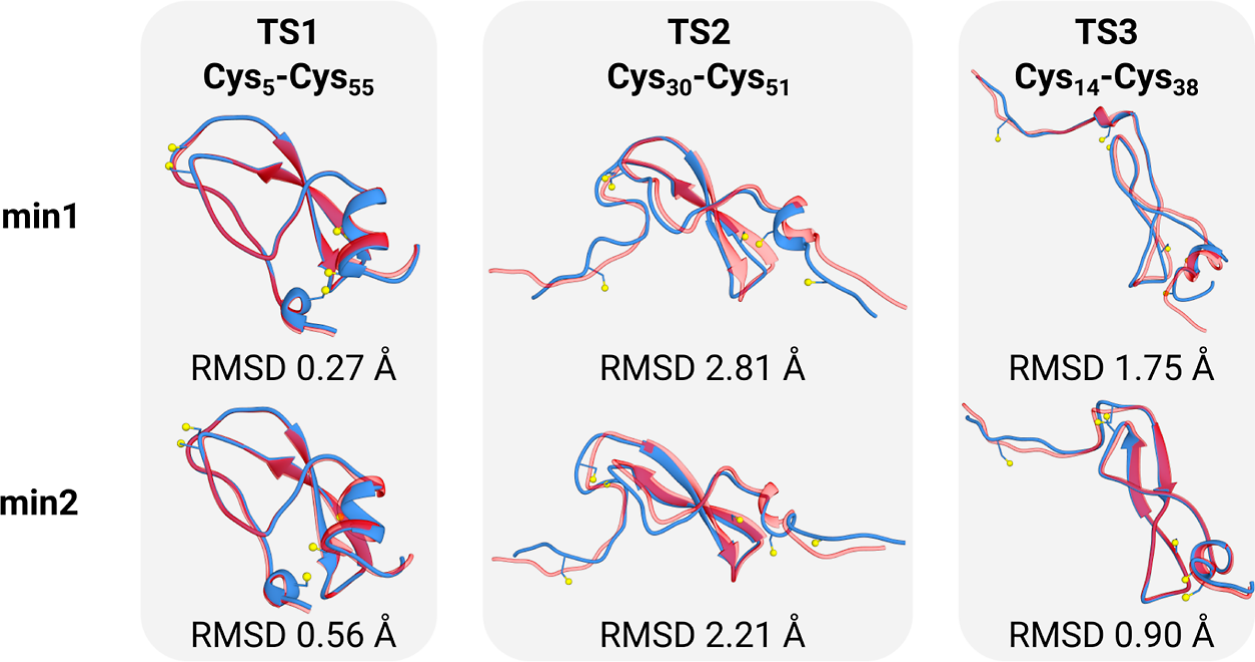
Comparison between the MC-ONIOM3 (solid blue) and GFN-FF (transparent red) reoptimized structures for the minima connected by transition states TS1, TS2, and TS3. “min1” denotes structures with intact disulfide bonds, and “min2” denotes structures with cleaved disulfide bonds. Heavy-atom rmsds are given below each structure pair. Positions for Cys-sulfur atoms have been marked for the MC-ONIOM3 geometries.

Further considerations must be made concerning the structural stability and conformational changes in the studied system due to the level of theory choice. Hence, Figure 6 provides a visual comparison between the MC-ONIOM3 and GFN-FF reoptimized structures for the minima associated with the paths TS1, TS2, and TS3. The heavy-atom root-mean-square deviations (rmsd) are displayed below each corresponding structure pair. This comparison allows for an assessment of the structural deviations between the two computational approaches.

rmsds underscore the crucial role of both noncovalent interactions and disulfide bonds in determining structural outcomes. The structure is well-defined by the disulfide bonds, as indicated by the remarkably low heavy-atom rmsd for the two minima connected by TS1. Energies and free energies calculated for these structures will be consistent across the different levels of theory and provide valid insights into the relative stabilities. The same is true for the minima linked by TS3, where residues are sufficiently separated, diminishing the influence of noncovalent interactions and leading to relatively small rmsds. Only in structures that remain relatively coiled, such as TS2, do numerous noncovalent interactions persist even after the breaking of two disulfide bonds. Here, the precision of the method to describe the noncovalent interactions significantly influences structural outcomes, resulting in substantial conformational differences (and high rmsds) between the force-field method and the ONIOM setup. Truncation of noncovalent interactions in the ONIOM layer can be an important source of error in this case,^32,33^ as was also seen for cystine in Section 3.1. Caution is advised when interpreting differences in stability for structures that differ significantly in terms of the noncovalent terms. The mismatches observed in the calculated stabilities of TS1/TS2 using MC-ONIOM3 (ΔΔ*G* of 32.6 kcal mol^–1^) versus the force-field (ΔΔ*G* of 9.6 kcal mol^–1^) probably stem from this factor.

**Figure 7:**
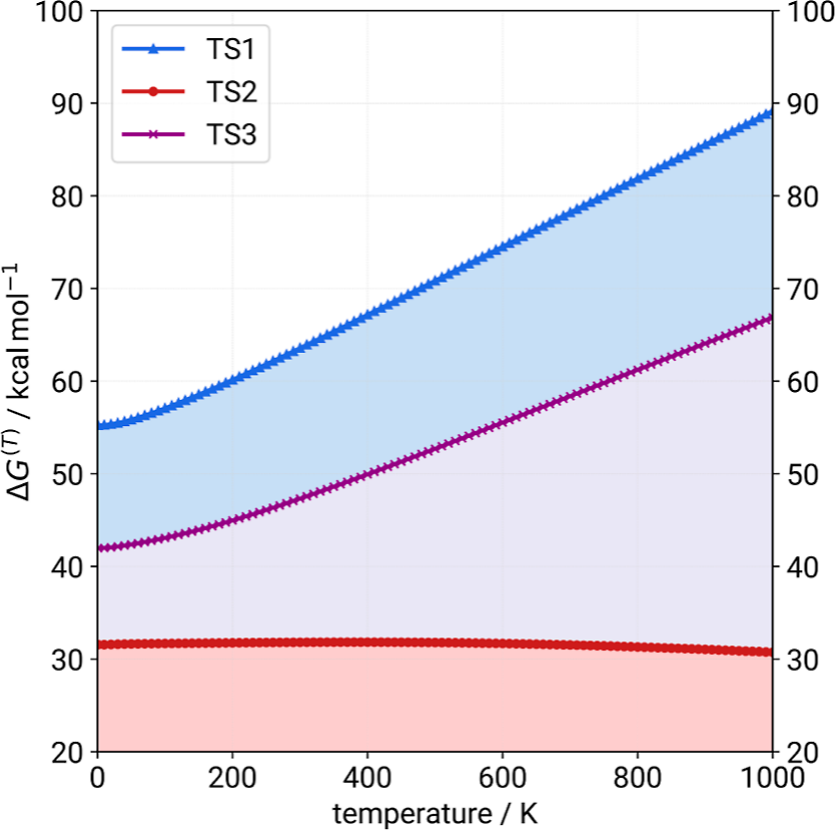
Reaction free energy (Δ*G*^(*T*)^) temperature dependence shown for the three disulfide bond cleavages of TS1 (Cys_5_–Cys_55_), TS2 (Cys_30_–Cys_51_), and TS3 (Cys_14_–Cys_38_).

Finally, Figure 7 illustrates the temperature dependence of the reaction free energy (Δ*G*^(*T*)^) for the three disulfide bond cleavages, calculated with MC-ONIOM3. This graphical representation provides valuable insights into the thermodynamic behavior of the reactions across a temperature range.

The overall positive Δ*G*^298.15^ values across all three reactions show the disulfide bond cleavage to be an endergonic process via the assumed oxidative reaction, as expected. For this type of reaction in the gas-phase, a further increase of the temperature is expected to increase the Gibbs free energy Δ*G*^(*T*)^, i.e., making the reaction even less likely to occur at higher temperatures. This trend was confirmed for TS1 and TS3, but surprisingly, TS2 instead exhibits an almost constant, or slightly decreasing, reaction free energy with rising temperature. Identifying a cause for this observation is nontrivial. However, examination of the individual contributions to Δδ*G*_mRRHO_ reveals a dominance of (vibrational) entropy with increasing temperature within the reaction free energies shown in Figure 7. Apparently, the two minima connected by TS2 have similar conformational flexibility, which leads to a stabilization of the reaction due to the respective entropy contributions. In the case of TS1 and TS3, the minima that contain intact disulfide bonds exhibit more low-frequency modes, contributing significantly more to the entropy than the connected minima with broken bonds. This result is consistent with chemical intuition and the expectation that a greater number of such modes with torsional character should be present for a more constrained but macrocyclic structure due to coupled internal rotations. We note, however, that in this representation, a part of the entropy, namely the landscape entropy associated with other minima, is missing because wider sampling is out of scope for the single-structure supramolecular approach employed for a system of this size.^58^ The final free energies might be affected by this omission, although we believe that it would not change the interpretation of the relative stabilities.

## 4 Conclusions

In this study, we employed the well-known ONIOM methodology to uncover the relative stability of disulfide bonds in the BPTI folding pathway and their associated energetics. A new standalone Fortran library called lwONIOM was introduced to enable multilayer and center ONIOM calculations, exploiting the all-atom general force-field GFN-FF, the semiempirical electronic structure method GFN2-xTB, and calculations at the r^2^SCAN-3c level of DFT. We provided insights into the computational strengths and limitations of these methods, in particular, investigating the robustness of GFN-FF in capturing structural changes and the ability to describe disulfide bond ruptures.

An initial exploration into the oxidative formation of cystine and the three disulfide bond cleavages encountered in BPTI revealed that GFN-FF does not accurately capture the required energetics. The reaction energies for the disulfide bonds, assessed at the MC-ONIOM3 level, exhibit a substantial stabilizing impact on the overall BPTI folding pathway, ranging from 34.4 to 55.6 kcal mol^–1^. Correspondingly, the associated Gibbs free energies, ranging from 30.9 to 63.6 kcal mol^–1^, highlight the significant influence of thermostatic contributions and their non-negligible effect at finite temperatures. These stabilities can be interpreted in agreement with experimental observations,^75^ although a true comparison will require us to model alternative Cys–Cys residue combinations and pathways. These pathways are not currently within the technical capabilities of GFN-FF, but we plan to address this limitation in future research. GFN-FF not only fails to accurately reproduce the relative stabilities of BPTI intermediates but also exhibits an incorrect sign. Intriguingly, a deliberate depiction of disulfide bond ruptures as homolytic dissociation, although chemically incorrect, manages to qualitatively reproduce the high-level energetics. This observation provides a good argument for utilizing force-field methods to survey folding pathways, subject to obvious caveats. The application of implicit solvation attenuates noncovalent interactions as expected but does not significantly affect the interpretation of relative disulfide stabilities.

In conclusion, we provide a qualitatively well-defined reference for the stability of disulfide bonds in biomolecular folding pathways. In future work, we aim to advance the computational methodology by directly running pathways and calculating transition states at the MC-ONIOM level. While the current project leveraged GFN-FF interfaced in the OPTIM program, the upcoming work will use a more direct implementation of the lwONIOM library. In this context, the investigation of (implicit) solvation effects and the integration of electrostatic rather than purely mechanical embedding in ONIOM are planned. To broaden the scope of our investigations and enable the simulation of even larger systems with higher accuracy, we are planning to update our library further by interfacing coarse-grained potentials, such as the UNited RESidue (UNRES) force-field,^78,79^ also recently interfaced to OPTIM.^21^ This integration will allow the use of composite QM/SQM/MM/CG methodologies. As we extend the capabilities of our computational tools, we hope that these advances will produce further insights into the dynamics of complex biomolecular systems.
